# Heat shock response and homeostatic plasticity

**DOI:** 10.3389/fncel.2015.00068

**Published:** 2015-03-12

**Authors:** Shanker Karunanithi, Ian R. Brown

**Affiliations:** ^1^School of Medical Science, Griffith UniversityQLD, Australia; ^2^Menzies Health Institute of Queensland, Griffith UniversityQLD, Australia; ^3^Department of Biological Sciences, Centre for the Neurobiology of Stress, University of Toronto ScarboroughToronto, ON, Canada

**Keywords:** adaptations, temperature, neuronal activity, Drosophila neuromuscular junction, synaptic homeostasis, action potentials and neuroprotection

## Abstract

Heat shock response and homeostatic plasticity are mechanisms that afford functional stability to cells in the face of stress. Each mechanism has been investigated independently, but the link between the two has not been extensively explored. We explore this link. The heat shock response enables cells to adapt to stresses such as high temperature, metabolic stress and reduced oxygen levels. This mechanism results from the production of heat shock proteins (HSPs) which maintain normal cellular functions by counteracting the misfolding of cellular proteins. Homeostatic plasticity enables neurons and their target cells to maintain their activity levels around their respective set points in the face of stress or disturbances. This mechanism results from the recruitment of adaptations at synaptic inputs, or at voltage-gated ion channels. In this perspective, we argue that heat shock triggers homeostatic plasticity through the production of HSPs. We also suggest that homeostatic plasticity is a form of neuroprotection.

## Introduction

The nervous system is particularly vulnerable to heat damage (Kourtis et al., 2012). However, it is well established that if organisms have been exposed to sublethal temperatures for brief periods (heat shock) and subsequently encounter lethal temperatures, their nervous systems will be protected from damage (Brown, 2007; Buccellato et al., 2007; Stetler et al., 2010; Robertson and Money, 2012). The protection afforded by heat shock is referred to as the heat shock response. Stressors other than elevated temperature, such as hypoxia, metabolic shock and hypothermia can also induce the heat shock response (Parsell and Lindquist, 1993; Gidalevitz et al., 2011; Morimoto, 2011). Therefore the response can be regarded as generally protective. The response occurs as a result of recruiting cellular adaptations during heat shock (Parsell and Lindquist, 1993; Latchman, 2004; Brown, 2007; Gidalevitz et al., 2011; Morimoto, 2011; van Oosten-Hawle and Morimoto, 2014). The response is found to be physiologically beneficial because it is induced within the mammalian nervous system following a fever-like increase in body temperature or through tissue injury (Brown, 2007; Asea and Brown, 2008).

Despite the existence of a large body of knowledge regarding the heat shock response, there is poor understanding of its protective effects on neuronal function. By reviewing the literature, we argue that the response contributes towards preserving a key functional parameter of neurons- neuronal activity- within their narrow physiological ranges at elevated temperatures.

## Homeostatic Plasticity

**Figure 1:**
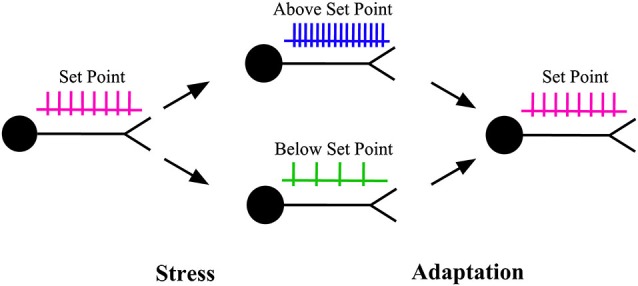
**Homeostatic plasticity**. When the activity level of a neuron is pushed above or below its set point (target level) by stress, a series of adaptations are recruited to restore the activity level back to its target level. The adaptations can be produced through changes in the properties of synaptic inputs to that neuron (homeostatic synaptic plasticity) or through changes in the properties of the ion channels that control neuronal excitability (homeostatic intrinsic plasticity).

In early life, neurons acquire their structural and functional characteristics which remain with them throughout the rest of their lifetime (Marder and Goaillard, 2006). An important problem that is at the forefront of research is to understand how neurons preserve their signature characteristics throughout their lifetime despite being subjected to stress, modification and rebuilding (Marder and Prinz, 2002; Marder and Goaillard, 2006). A key characteristic that is thought to be tightly regulated throughout a neuron’s lifetime is neuronal activity or the action potential firing rate, which carries information for producing behaviors (Marder and Goaillard, 2006; Debanne et al., 2011; Bishop and Zito, 2013; Hengen et al., 2013; Keck et al., 2013). The preservation of neuronal activity around a set point (target level) is thought to be key to the maintenance of nervous system function in the face of stress, insults or disturbances (Marder and Prinz, 2002; Turrigiano and Nelson, 2004; Turrigiano, 2008; Davis, 2013; Figure 1). The mechanism that preserves neuronal activity around a set point is referred to as homeostatic plasticity (Turrigiano and Nelson, 2004; Davis, 2013).

Research over the past 20 years indicates that homeostatic plasticity can result from adaptations which alter the strengths of synapses or the properties of ion channels (other than postsynaptic ligand-gated channels) that control neuronal excitability, or both (Turrigiano, 1999, 2012; Marder and Prinz, 2002; Turrigiano and Nelson, 2004). The adaptations at synapses are referred to as homeostatic synaptic plasticity, whereas those at ion channels are referred to as homeostatic intrinsic plasticity (Desai, 2003; Misonou, 2010).

## Neuroprotection and Homeostatic Plasticity

Neuroprotection refers to the preservation of neuronal structure or function, or both, against insults (Casson et al., 2012). Up to now, the heat shock response has been considered as a mechanism that affords neuroprotection (Brown, 2007). Whether the response can also be considered as a mechanism that affords homeostatic plasticity has not been explored (Davis, 2006; Brown, 2007; Robertson and Money, 2012). Interestingly, homeostatic plasticity appears to fall within the definition of neuroprotection because it describes the preservation of neuronal function against insults. By reviewing publications investigating the role of heat shock on neuronal function, we argue that the effects of heat shock, which are considered to be neuroprotection could also be homeostatic plasticity. Specifically, we will examine the heat shock response at elevated temperatures.

## Heat Shock Proteins

When organisms receive a heat shock, the synthesis of most cellular proteins is downregulated with the exception of a small class of proteins, which includes HSPs (Parsell and Lindquist, 1993; Morimoto, 2011). HSPs maintain cellular integrity by preventing other cellular proteins from unfolding or degrading, or preventing unfolded proteins from aggregating (Gidalevitz et al., 2011). HSPs are classified according to their molecular mass (for example, HSP40 represents the HSP that is 40 kDa in mass) and grouped into the following families: HSP100, HSP90, HSP70, HSP40, small HSPs and chaperonins (Kampinga et al., 2009; De Maio and Vazquez, 2013).

HSPs that are present in unstressed cells are referred to as constitutive HSPs or molecular chaperones whereas those that are expressed following stress are referred to as stress proteins or induced HSPs (De Maio, 1999; De Maio and Vazquez, 2013; Saibil, 2013). Constitutive HSPs, such as HSP90, HSP40 and HSC70 perform housekeeping functions within cells (De Maio, 1999; Hartl and Hayer-Hartl, 2009; Saibil, 2013). Induced HSPs, such as HSP70 (which is the most abundantly induced HSP following heat shock), are involved in folding proteins correctly and preventing the aggregation of unfolded proteins (De Maio, 1999; Saibil, 2013). In brain cells, hyperthermia triggers a robust expression of induced HSPs, such as HSP70, HSP32 and HSP27 (Brown, 2007; Asea and Brown, 2008).

## Heat Shock and Homeostatic Synaptic Plasticity

Synapses are the critical sites of cell-to-cell communication within the nervous system. To prevent communication breakdown under stressful conditions, synapses need to remain functional (Karunanithi et al., 1999, 2002). Heat shock is shown to be beneficial in preserving synapse function at elevated temperatures (Freedman et al., 1981; Dawson-Scully and Meldrum Robertson, 1998; Karunanithi et al., 1999, 2002; Kelty et al., 2002; Klose et al., 2004, 2008; Newman et al., 2005). However, the underlying mechanisms have not been extensively explored. In this section, we will review the effects of heat shock on synapse function and suggest that the observed changes could be homeostatic synaptic plasticity.

An important parameter that represents the effectiveness of synaptic transmission is synaptic strength. Synaptic strength is defined as the average size of the postsynaptic responses produced upon nerve stimulation at low frequencies, or the response on initial stimulation during a train of stimuli (Atwood and Karunanithi, 2002). In homeostatic synaptic plasticity, synaptic strengths are adjusted in a manner that lead to the preservation of neuronal activity around a set point (Turrigiano, 1999, 2011; Burrone and Murthy, 2003). In some cases of homeostatic synaptic plasticity, synaptic strengths are maintained at a constant value. This form of plasticity is referred to as synaptic homeostasis (Stewart et al., 1996; Turrigiano, 1999; Davis, 2013; Wang et al., 2014; Davis and Müller, 2015).

**Figure 2:**
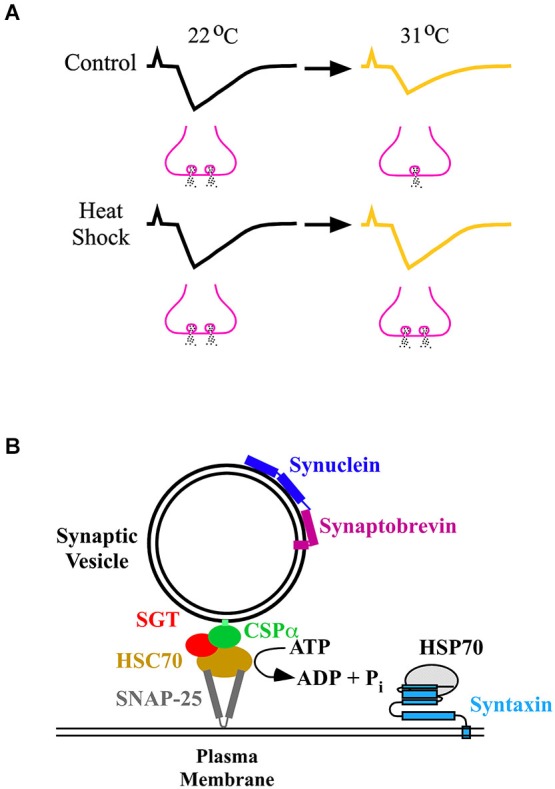
**(A)** Synaptic homeostasis following heat shock. In control *Drosophila* larvae that did not receive a prior heat shock, synaptic strength (upper traces) decreased at 31°C compared to room temperature (22°C) through a decrease in neurotransmitter release (fewer vesicles releasing neurotransmitter). Following heat shock, synaptic strength (lower traces) at 31°C was maintained at the same value as that observed at 22°C by preventing a decrease in neurotransmitter release (the same number of vesicles releasing neurotransmitter). **(B)** Induced HSPs and chaperones neuroprotect the neurotransmitter release machinery. The diagram shows the chaperones and some of the proteins which constitute the release machinery (Rizo and Südhof, 2012). Chaperones prevent proteins from unfolding, or engaging in unwanted interactions, or both. They maintain proteins that constitute the neurotransmitter release machinery in a release-ready state before calcium-triggered synaptic vesicle exocytosis. The CSP/HSC70/SGT (small glutamine-rich protein) complex chaperones SNAP-25, and synuclein chaperones synaptobrevin, maintaining SNAP-25 and synaptobrevin, respectively, in a release-ready state. Upon release from the chaperones, synaptobrevin rapidly assembles with SNAP-25 and syntaxin, forming the SNARE complex that leads to exocytosis. The CSP complex operates in an ATP-dependent manner, whereas synuclein operates in an ATP-independent manner. HSP70 is reported to interact with syntaxin (Fei et al., 2007). HSP70 could protect syntaxin from becoming compromised under stress, enabling syntaxin to form the SNARE complex and undergo exocytosis.

Current findings indicate that synaptic homeostasis leads to the preservation of muscle activity around a set point (Davis, 2006, 2013). At the *Drosophila* larval neuromuscular junction (NMJ), synaptic strengths were attenuated above room temperature (22°C) (Karunanithi et al., 1999). However, *Drosophila* larvae that received a prior heat shock displayed synaptic homeostasis, where synaptic strengths were maintained up to temperatures 9°C higher than room temperature (Karunanithi et al., 1999; Figure 2A). Heat shock also afforded synaptic homeostasis at the locust NMJ (Dawson-Scully and Meldrum Robertson, 1998; Barclay and Robertson, 2000). These findings indicate that heat shock affords synaptic homeostasis at elevated temperatures, preventing decreases in the levels of synaptic excitation of the muscle.

What are the factor(s) which heat shock protects to afford synaptic homeostasis? At the *Drosophila* larval NMJ, the most significant factor which heat shock protected at elevated temperatures was nerve-evoked neurotransmitter release (Karunanithi et al., 1999, 2002; Figure 2A). An absolute requirement for nerve-evoked neurotransmitter release is calcium entry into nerve terminals through voltage-gated calcium channels (VGCCs; Macleod et al., 2006). Results indicate that the preservation of neurotransmitter release at elevated temperatures following heat shock may partly result from the preservation of calcium entry through VGCCs (Klose et al., 2008).

Overwhelming evidence indicates that homeostatic synaptic plasticity is calcium dependent (Pozo and Goda, 2010; Turrigiano, 2012). At heat shocked *Drosophila* larval NMJs, intracellular calcium-handling mechanisms that contribute toward sustaining neurotransmitter release at elevated temperatures were protected (Klose et al., 2008, 2009). Resting calcium concentration and calcium clearance were maintained above the upper temperature limit of the organism (Klose et al., 2008). Protection of such calcium-handling mechanisms following heat shock may lead to greater availability of calcium to the release machinery to sustain neurotransmitter release at elevated temperatures (Barclay and Robertson, 2003; Klose et al., 2008). The available data suggests that these mechanisms may contribute towards affording synaptic homeostasis at elevated temperatures.

Following heat shock, which molecules are responsible for establishing synaptic homeostasis? The extensive work of Brown et al. has shown that following heat shock, HSPs localize at mammalian synapses (Freedman et al., 1981; Bechtold and Brown, 2000, 2003; Bechtold et al., 2000; Chen and Brown, 2007; Asea and Brown, 2008). Existing literature strongly argues that HSPs afford protection to synapses, especially HSP70. Studies have shown that HSP70 is rapidly induced by heat shock (Tissières et al., 1974; Parsell and Lindquist, 1993; Feder et al., 1996). In heat shocked *Drosophila* larvae, synaptic strength at the larval NMJ paralleled the levels of HSP70 expressed in the organism (Karunanithi et al., 1999, 2002). Targeting HSP70 to *Drosophila* motor neurons induced structural plasticity of axonal terminals resulting in increased neurotransmitter release at elevated temperatures at the larval NMJ (Xiao et al., 2007). Presynaptic calcium entry, resting calcium concentrations and calcium clearance were uncompromised at elevated temperatures when HSP70 was overexpressed into motor neurons (Klose et al., 2008). HSP70 is also thought to protect calcium-handling proteins, such as sarco-endoplasmic reticulum calcium-ATPase (SERCA; Klose et al., 2008). SERCA regulates endoplasmic reticulum calcium stores to control intracellular calcium concentrations (Klose et al., 2008). These results suggest, that following heat shock, induced HSP70 sustains neurotransmitter release at elevated temperatures to afford synaptic homeostasis (Fei et al., 2007, 2008; Xiao et al., 2007).

In *Drosophila* mutant larvae that fail to produce induced HSPs following heat shock, there was a compensatory upregulation of constitutive HSPs (Neal et al., 2006). This upregulation contributed to the preservation of synaptic function at elevated temperatures (Neal et al., 2006). Presynaptic proteins other than HSPs, such as cysteine string protein (CSP) and α-synuclein, have also been reported to protect the transmitter release machinery from failing (Burré et al., 2010; Sharma et al., 2011). CSP is found to afford protection by interacting with constitutive HSPs, such as HSC70 (Bronk et al., 2001, 2005; Sharma et al., 2011; Figure 2B). These results suggest that proteins other than induced HSPs could also prevent failure of neurotransmitter release.

## Heat Shock and Homeostatic Intrinsic Plasticity

When organisms are in danger or under stress, their nervous systems need to be able to produce action potentials in order to help them generate escape behaviors (such as behaviors that enable organisms to escape from prey or from the heat on hot days) (Barclay and Robertson, 2000; Money et al., 2005; Feder et al., 2010). The collective evidence indicates that heat shock alters the properties of voltage-gated ion channels in order to sustain action potential firing at elevated temperatures.

In motor neurons that control locust flight muscles, heat shock increased the upper temperature limit at which action potentials could fire, preventing action potential failure at elevated temperatures (Wu et al., 2001). Decreases in K^+^ currents increased action potential duration to prevent action potential failure at elevated temperatures (Wu et al., 2001). In locust motor neurons and visual interneurons, heat shock produced an increase in action potential amplitude at elevated temperatures (Wu et al., 2001; Money et al., 2005). That effect could be due to heat shock preventing the inactivation of voltage-gated Na^+^ channels at elevated temperatures (Money et al., 2005). These results indicate that heat shock promotes a form of homeostatic intrinsic plasticity where the conductance of voltage-gated Na^+^ and K^+^ channels are altered to enable action potential firing at elevated temperatures.

HSP interactions with voltage-gated ion channels that aid action potential firing are poorly understood. In previous work, HSPs were found to interact with VGCCs (Krieger et al., 2006) and voltage-gated potassium channels (Ramirez et al., 1999; Clay and Kuzirian, 2002; Ficker et al., 2003; Wray, 2009; Gao et al., 2013a,b). In HEK cells, HSP70 was proposed to aid the targeting of protein kinase C to specific regions of the voltage-gated Ca*_v_*2.3 calcium channel (Krieger et al., 2006). K*_v_*7.4 voltage-gated potassium channels are found on the outer hair cell membranes of the inner ear and are involved in the conduction of sound (Gao et al., 2013a,b). Overexpression of HSP90 was found to prevent the loss of surface K*_v_*7.4 channels on HEK cells (Gao et al., 2013a,b), suggesting that overexpression of HSP90 could prevent hearing loss. These findings indicate that HSPs could potentially interact with voltage-gated ion channels to induce homeostatic intrinsic plasticity.

Studies have shown that there are additional benefits afforded by heat shock in the reliable generation of action potentials at elevated temperatures (Money et al., 2005, 2009; Hou et al., 2014). First, there was increased hyperpolarization of the membrane to maintain action potential amplitude. At elevated temperatures, there is a build up of extracellular K^+^ that depolarizes the membrane, preventing the generation of action potentials. Heat shock prevented this build up by causing the clearance of extracellular K^+^ through insertion of more Na^+^/K^+^-ATPase pumps into the cell membrane. These pumps are designed to move K^+^ into the cell and this movement probably contributed to the hyperpolarization (Money et al., 2009; Hou et al., 2014). The idea that active transporters can alter membrane excitability is further supported by recent work showing that in *Drosophila* larval motor neurons, changes in Na^+^/K^+^-ATPase pump function can lead to long-lasting changes in membrane excitability (Pulver and Griffith, 2010). Second, there was increased membrane excitability to enable generation of high frequency bursts of action potentials (Money et al., 2005). For example, heat shock has been shown to increase membrane excitability by increasing the afterdepolarizations following an action potential. The afterdepolarizations lower the threshold for subsequent action potential firing. Prolonging the conductance of Na^+^, or Ca^2+^ or another non-selective cation channel is thought to produce these afterdepolarizations. Alternatively, a reduction in K^+^ conductance could be another means for generating afterdepolarizations (Money et al., 2005). These results indicate that heat shock may recruit homeostatic intrinsic plasticity through alterations in not just the properties of voltage-gated ion channels, but also those of active-transporters.

Early work from the Tytell laboratory conducted on the squid giant axon showed that heat shock caused HSP70 to be rapidly synthesized and transferred from the surrounding glia into the axon (Tytell et al., 1986). This glia to neuron transfer may provide a mechanism for fast delivery of HSPs that may play a functional role in axons (Armstrong et al., 2011). Previous work has shown that HSP70 promotes the anchoring of Na^+^/K^+^-ATPase pumps to the cytoskeletal network located just beneath the neuronal membrane (Riordan et al., 2005; Ruete et al., 2008). By preventing the loss of such pumps from the membrane, the build up of extracellular K^+^ at elevated temperatures could be reduced to promote membrane hyperpolarization and action potential firing. In another example, hyperthermia caused the production of HSP27 and HSP32 in perisynaptic glial cells which surround synapses. These HSPs were then transferred into the synapses to potentially afford them protection (Bechtold and Brown, 2003). These results suggest that the transfer of HSPs into neurons from glia provides neuroprotection and could be a means of preventing action potential failure under stress.

## Heat Shock and Homeostatic Plasticity

It is thought that if homeostatic plasticity preserves the normal activity of individual neural circuit elements, the circuit itself should be afforded functional stability (Turrigiano, 2011, 2012). Observations in the locomotory systems of *Drosophila* and locusts suggest that heat shock affords functional stability to neural circuits under stress by recruiting homeostatic plasticity. Motor patterns are rhythmic bursts of electrical activity that are generated by motor circuits to drive movements (Barclay et al., 2002). Previous work has shown that motor patterns were compromised at elevated temperatures in *Drosophila* and locusts (Robertson et al., 1996; Klose et al., 2005; Robertson and Money, 2012). However, this is prevented if they receive a prior heat shock (Robertson et al., 1996; Klose et al., 2005). In *Drosophila*, previous work has shown that, by overexpressing HSP70 in motor neurons, synaptic performance is improved at elevated temperatures, protecting larval locomotion (Xiao et al., 2007). Therefore it appears that a prior heat shock can protect neural circuit function at elevated temperatures. It remains to be determined whether this occurs by homeostatically maintaining the normal activity of individual neural circuit elements.

## Concluding Remarks

Heat shock response is a mechanism that preserves cellular functions under stress through the upregulation of HSPs. Homeostatic plasticity is a mechanism that preserves the activities of neurons and their target cells around their respective set points in the face of stress. Therefore we propose that the heat shock response may afford homeostatic plasticity. In this review, we have sought to provide examples from literature suggesting that the heat shock response affords homeostatic plasticity. Currently, investigations of the link between the two mechanisms are scant. By providing such examples, we hope to stimulate the interest of readers to further explore the interface between these two mechanisms.

Certain disorders, such as seizures, sleep disturbances and cognitive disorders are thought to result, in part, from disruptions of homeostatic plasticity (Burke and Barnes, 2010; Wang et al., 2011; Huguet et al., 2013; Swann and Rho, 2014; Tononi and Cirelli, 2014). HSPs have been shown to ameliorate the symptoms of these disorders (Shaw et al., 2002; Naidoo et al., 2008; Su et al., 2009; Ekimova et al., 2010; Vizcaychipi et al., 2011; Hashimoto-Torii et al., 2014). Whether the ameliorative effects of the heat shock response are produced through the recruitment of homeostatic plasticity will be a fascinating field of study that may have clinical implications.

## Conflict of Interest Statement

The authors declare that the research was conducted in the absence of any commercial or financial relationships that could be construed as a potential conflict of interest.
